# Large‐Scale Transdisciplinary Collaboration for Adaptation Research: Challenges and Insights

**DOI:** 10.1002/gch2.201700132

**Published:** 2018-05-02

**Authors:** Georgina Cundill, Blane Harvey, Mark Tebboth, Logan Cochrane, Bruce Currie‐Alder, Katharine Vincent, Jon Lawn, Robert. J. Nicholls, Lucia Scodanibbio, Anjal Prakash, Mark New, Philippus Wester, Michele Leone, Daniel Morchain, Eva Ludi, Jesse DeMaria‐Kinney, Ahmed Khan, Marie‐Eve Landry

**Affiliations:** ^1^ International Development Research Centre Ottawa K1P 0B2 Canada; ^2^ Department of Integrated Studies in Education McGill University Montreal H3A 2T5 Canada; ^3^ School of International Development Tyndall Centre for Climate Change Research University of East Anglia Norwich NR4 7TJ UK; ^4^ Global and International Studies Carleton University Ottawa K1P 0B2 Canada; ^5^ Kulima Integrated Development Solutions Pietermaritzburg 3200 South Africa; ^6^ Faculty of Engineering and the Environment University of Southampton SO17 1BJ UK; ^7^ African Climate and Development Initiative University of Cape Town Cape Town 8001 South Africa; ^8^ International Centre for Integrated Mountain Development Kathmandu Nepal; ^9^ African Climate and Development Initiative University of Cape Town Cape Town 8001 South Africa; ^10^ School of International Development Tyndall Centre for Climate Change Research University of East Anglia Norwich NR4 7TJ UK; ^11^ International Development Research Centre Nairobi Kenya; ^12^ Oxfam GB Oxford OX4 2JY UK; ^13^ Overseas Development Institute London SE1 8NJ UK

**Keywords:** climate change, collaboration, transdisciplinarity

## Abstract

An increasing number of research programs seek to support adaptation to climate change through the engagement of large‐scale transdisciplinary networks that span countries and continents. While transdisciplinary research processes have been a topic of reflection, practice, and refinement for some time, these trends now mean that the global change research community needs to reflect and learn how to pursue collaborative research on a large scale. This paper shares insights from a seven‐year climate change adaptation research program that supports collaboration between more than 450 researchers and practitioners across four consortia and 17 countries. The experience confirms the importance of attention to careful design for transdisciplinary collaboration, but also highlights that this alone is not enough. The success of well‐designed transdisciplinary research processes is also strongly influenced by relational and systemic features of collaborative relationships. Relational features include interpersonal trust, mutual respect, and leadership styles, while systemic features include legal partnership agreements, power asymmetries between partners, and institutional values and cultures. In the new arena of large‐scale collaborative science efforts, enablers of transdisciplinary collaboration include dedicated project coordinators, leaders at multiple levels, and the availability of small amounts of flexible funds to enable nimble responses to opportunities and unexpected collaborations.

A growing number of large‐scale climate change adaptation research programs have emerged in recent years to generate knowledge that contributes toward impact and change. Examples include the Collaborative Adaptation Research Initiative in Africa and Asia (CARIAA), Future Climate for Africa, and Ecosystem Services for Poverty Alleviation. It is imperative that we treat these emerging collaborative endeavors as experiments from which we can actively learn about how to pursue transdisciplinary collaboration in ways that support impact at appropriate scales.

In this paper, we offer a commentary from one of these programs, CARIAA, where significant and purposeful investment was made into the design for transdisciplinary collaboration. The CARIAA experience suggests that it is critical to pay attention to three key dimensions of transdisciplinary collaboration in large‐scale research programs. These are design, or how programs are structured to support collaboration and impact; relational features, or how interpersonal and interinstitutional dynamics evolve and are mediated; and systemic features, which refer to pre‐existing norms and biases that affect how the other two dimensions take shape. Our experience also highlights the importance of built‐in flexibility in the availability of funds to enable emergent collaboration beyond initial expectations.

The shift toward large scale transdisciplinary collaborations in global change research has come about for a variety of reasons. These reasons include the scale and urgency of environmental challenges such as climate change, a growing appreciation of the complexity of the problems, and therefore the need for learning‐oriented approaches to tackling them, and shifting realities in how publicly funded research is justified by public agencies.

Climate action, specifically, requires integrated and multiscale research that is simultaneously cutting edge, problem‐oriented, and that creates space for other ways of knowing, beyond western science alone.^1^ Transdisciplinary research, unsurprisingly, has come to fore in this environment.^2^ Transdisciplinary research refers to research processes that support mutual learning across disciplinary divides and knowledge domains, with the goal of producing shared knowledge around a common problem.^3^ A central feature of transdisciplinary approaches is collaboration and mutual learning among diverse stakeholders who share a commitment to tackling complex social and ecological problems.^4^ The intention is often to coproduce new understandings across a diversity of knowledge domains, thereby enhancing the possibility of transformative change.^5^ However, while early development of transdisciplinary theory was somewhat preoccupied, with good reason, with the integration of knowledge (e.g., ref. 6), today our challenges tend to rotate around the more pragmatic questions of how to operationalize collaboration between individuals from research, policy, and civil society sectors, for impact, in geographically and culturally distant teams.

These questions have come to the fore as the complexity and urgency of the environmental challenges that we face have become more apparent, which has in turn highlighted the scale of response required, and partially in response to significant shifts in the research funding environment. Rising demands for science funding within the research community, and rising scrutiny of public spending by civil society, have led a number of funders to become more prescriptive in identifying research priorities and desired outcomes.^7^ One trend has been to steer funding toward addressing “societal challenges,” which imply three things: high stakes issues, phenomenon covering a wide geographic scale, and a scope of effort that exceeds the capacity of individual organizations or nations working in isolation.^8^ To justify public spending on science, funders have therefore tended to narrow the research agenda to a limited set of problems and larger‐scale grants that increase the ambition regarding what science is expected to deliver for society. Where previous funding programs were deemed a success by generating new knowledge, today research funding is often expected to provide solutions to the problems that society faces, and even take steps toward putting those solutions into action.

The “team science” literature has generated a strong understanding of a variety of factors that either enable or constrain research collaborations.^9^ Many of these features are now common knowledge to most researchers working collaboratively in the field of climate change (**Table** **1**). Building out from this work, scholars working more specifically on transdisciplinary collaboration have focused on process design principles that help deal with these well‐known enabling and constraining factors. Design‐oriented efforts have provided conceptual frameworks offering phased approaches that emphasize shared problem framing, team building, and the cocreation of solution‐oriented knowledge,^10^ the identification of empirically derived lessons directed at fostering learning in large‐scale transdisciplinary endeavors,^11^ and the identification of principles that can support constructive dialogue.^12^ Frequently, attention is paid to the personal dispositions needed for individuals to take part in such processes.^13^

**Table 1:** Enablers and constraints of collaboration in research environments (adapted from Stokols^14^)

Enablers	Constraints
Frequent face‐to‐face interactions	Significant time requirements to establish common conceptual frameworks and personal relationships
Interpersonal skills of team leaders	Conflicts among different knowledge systems with regard to both research process and valid knowledge
Commitment to achieving transdisciplinary goals and outcomes	Bureaucratic barriers between departments and institutions
A history of prior relationships among team members and organizations	Perceived status differences among academic and nonacademic partners
Spatial proximity of team members	Unrealistic expectations about shared goals and products
Easy‐to‐use electronic linkages between distant team members	Language differences
Maintaining continuity of individuals from the start to end of a project	Competing or conflicting organizational priorities
Actively dealing with perceived status differences between the academic and nonacademic partners	

While a great deal of attention has been paid to these necessary design features for transdisciplinary collaboration, and to the personal dispositions of individuals that influence their ability to make the most of well‐designed processes, less attention has been paid to the relational and systemic barriers that can profoundly undermine the best process design and the best personal intentions. Recently, in a review of 41 transdisciplinary case studies, Scholz and Steiner^15^ showed that a large number of barriers may be encountered due to either the context in which a transdisciplinary process unfolds, or during different phases of a project, for example, from inception through to close‐down. Drawing on experiences in CARIAA, we illuminate some of the relational and systemic barriers that emerged as particularly important for large‐scale transdisciplinary collaboration throughout that program.

The CARIAA program has sought to support better‐informed policy and practice in climate change hotspots in Africa and Asia. A climate change hotspot is an area where climate change is expected to impact regions with a high concentration of particularly vulnerable people.^16^ The research program therefore faced a complex and urgent problem that manifested and required responses at multiple scales. Given the focus on vulnerable populations, the program also placed a strong emphasis on research teams demonstrating impact as a result of their work.

Planning for transdisciplinary collaboration was therefore part of the very earliest design of the program. As the managing partner of the CARIAA program, the International Development Research Centre (IDRC) invested ≈50% of the program management budget in process design and programmatic leadership to support transdisciplinary collaboration among some 450 researchers and practitioners from more than 40 organizations. The program is organized into four consortia, whose members come from research, policy, and civil society organizations and collaborate around their own common programs of work for a specific hotspot environment in different regions. Explicit focus was placed on research uptake from the start of the program, with consortia required to develop strategies for getting research evidence into use and encouraged to develop transdisciplinary partnerships with nonacademic partners to this end.

CARIAA drew on critical reflections from others to design explicit plans to foster collaboration and mutual learning between the partners involved (explicitly drawing, for example, on ref. 17) The aim was to create collaborative spaces embedded in ongoing mutual learning processes (these are shown in **Figure** **1**), which would result in a variety of synthesis outputs and impacts, representing knowledge integration from a variety of sources. These various collaborative spaces were embedded within a learning framework. The learning framework aimed at supporting mutual learning and included annual learning reviews where members of all four consortia convened around common themes and problem domains (for example, climate induced migration), mid‐year learning reviews for research uptake specialists, thematic webinars, and a mid‐term formative evaluation of the whole program. At the program level, all face‐to‐face learning engagements (such as annual meetings) were codesigned with members of the four consortia and the program management team, with external professional facilitation support. Importantly, the program team also built flexibility into budget allocations to allow for adaptive management in response to new learning about how collaboration could be fostered. A key element of this was an “Opportunities and Synergies Fund,” which are flexible funds intended to support emergent and unplanned collaborations.

**Figure 1:**
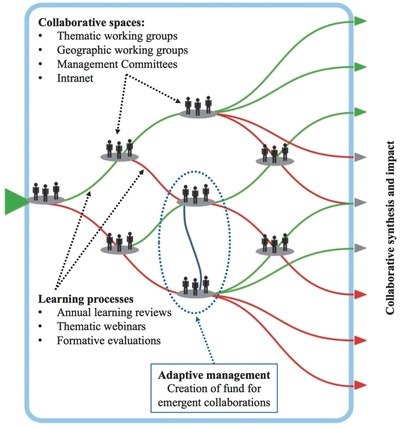
Investments in process design for transdisciplinary collaboration in CARIAA (image adapted from Figure SPM.9 (B) from ref. 18).

Similarly, the individual consortia included a number of design elements aimed at fostering transdisciplinary collaboration, and specifically mutual learning around common problems. While some consortia organized themselves into regional teams that would collaborate around regional priority research and policy questions, others designed their projects into work packages that cut across regions and/or scales. One of the consortia used a standardized research methodology to collect data across all research sites, with the intention of supporting collaborative synthesis of findings and the development of shared purpose. Another mirrored the Opportunities and Synergies Fund at a program level and created a Small Opportunities Grant within their consortium, enabling early career researchers and practitioners to engage with more senior individuals in other institutions around a specific issue. Common across all consortia was a targeted approach to research uptake and impact, and an effort to forge partnerships beyond research organizations. This emphasis on pursuing impact and change was inserted by design, but was modified and adapted throughout the duration of the program. While in one consortia research uptake efforts were led by an international non‐governmental organization (NGO) with support from local NGOs and practitioners, in others it was led by researchers.

The heavy investment in supporting mutual learning as a core element of transdisciplinary collaboration^15^ across all scales of CARIAA, has provided insight into the underlying enablers and constraints of transdisciplinary collaboration on a large scale. These design features of the CARIAA program have been described in depth elsewhere.^11^ While design features have been crucial to the successes in the program, we have learned that a handful of relational and systemic features underlie these design elements, and must be recognized in efforts to support transdisciplinary collaboration (**Figure** **2**). As stated at the outset of the paper, relational features refer to the ways in which interpersonal and inter‐institutional interactions evolve and are mediated, while systemic features refer to pre‐existing norms and biases that affect how relational features of transdisciplinary collaborative endeavors take shape. We discuss these relational and systemic features in turn below.

**Figure 2:**
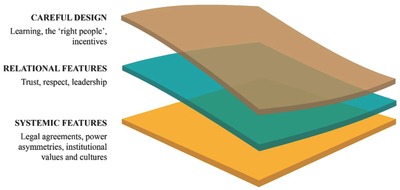
The layered features of transdisciplinary collaboration.

Like other large‐scale programs, CARIAA has a number of key features that make trust a foundational issue that, if ignored, can undermine all other investments into design for collaboration. First, consortia often comprise partners who have not worked together before. Second, consortium members tend to be geographically dispersed and interact virtually rather than face‐to‐face (Table 1). Third, highly competitive funding models bring together individuals and institutions with long histories as competitors, rather than collaborators. These same organizations may continue to compete for other sources of funding despite collaborating within one program. Competitive funding models may also create incentives to keep skills, knowledge, and unpublished data internal, as opposed to what is expected in collaborative arrangements.^19^ It is often unrealistic to expect these dynamics to radically shift in a short period of time into an open and trusting relationship.

In multicountry consortia, particularly in South–North partnerships, relationships between individuals and institutions are influenced by culturally and historically constituted notions of respect. A higher ranked university may resent, for example, being managed (as a subcontractor) by a lower ranked university. Similarly, deep‐seated historical injustices, perceptions, and experiences of cultural domination and economic dependencies can simmer just below the surface of any relationship, rendering attempts to create incentives and positive learning environments ineffective. Accepting what can, and what cannot, be changed during a five‐year research program is crucial for learning how to navigate these deep structural barriers to transdisciplinary collaboration. It is critically important to carefully consider the ways in which the structure of partnerships may serve to reinforce such perceptions and experiences from the inception phase of any collaboration.

Leadership is a relational feature of collaboration because it emerges from the relationship between individuals in a shared endeavor. The most successful leadership styles in CARIAA have been characterized as inclusive and hands‐on, drawing partners into project planning and design, ensuring that their interests and ideas are incorporated into work streams, and that they have a real stake in the outcomes. In some cultural contexts, success arose where leaders considered friendships, and not purely professional relationships, as an important part of collaborative endeavors. In some cases, such friendships made missing incentives less important in critical decisions about whether or not a given partner would collaborate on a task or activity, in other instances, such friendships constituted the incentive to collaborate.

Trust, respect, and leadership are all relational features of teams. They are concerned with how people interact and respond to one another, and about people's positionality in relation to others. These are not personal features of individuals, they emerge through interaction with others, and are therefore harder to factor into carefully designed transdisciplinary collaborative processes.

However, even when trust, appropriate leadership, and careful investment in design for collaboration come together in a transdisciplinary team, the CARIAA experience suggests that a handful of systemic enablers and constraints can still strongly influence outcomes. These systemic features include the design of partnership processes and agreements, the presence of power asymmetries between partners, and conflicting institutional cultures, values, and understandings of success.

In large research programs, risk management is essential to ensure that funds are spent effectively. However, risk‐management strategies can lead to legally binding partnership agreements that undermine collaborative outcomes. In CARIAA, an important risk management strategy was to develop individual grant agreements between the IDRC and the core partners in each of the four consortia. The expectation, however, was that partners would collaborate both within and between the consortia. The presence of individual grant agreements provided an unanticipated disincentive for core partners to collaborate, since they reported to the funder individually, rather than collectively.

Some consortia solved this challenge for themselves by creating “Research Collaboration Agreements” that tied the consortium partners together legally, with Terms and Conditions, in a peer level agreement. These agreements ensured that the partners were clear on core partnership values and expectations, on acceptable means of interaction, and that they were accountable to one another. Another novel solution, from a contractual perspective, was to create the flexible small grants funds described above for both the program overall and in one of the consortia. These flexible small grants allowed a balance between risk management and the agility needed to pursue opportunities and collaborative arrangements unforeseen at the start of the program.

Transdisciplinary collaborations inevitably bring together partners who hold different levels of power, as perceived by themselves and others, in a variety of domains. Some partners will be more powerful by virtue of their mandate (e.g., a multilateral organization versus a local research organization), while others may hold a privileged position in terms of the respect that their knowledge commands (a university compared with a civil society organization), and still others will hold power because of their command of the English language. There are also individual power differences (based on educational background, gender and age, among others) that play out in different cultural contexts. Such power differentials can act as a barrier to effective collaboration and transdisciplinary learning.

One consortium dealt with the fact that they had a particularly dominant institution in the lead by contracting an independent partnership broker to lead a participatory process of developing partnership principles and operational guidelines for all partners. These internal agreements were signed by all partners, and were frequently returned to during the project in order to resolve disagreements. This process was not easy, and came with high transaction costs. On the whole, however, the approach was positive and necessary given the power asymmetries at play. Our experience shows that power asymmetries are a deep systemic barrier, however they can and should be navigated (as demonstrated above) rather than approached with a naive expectation that perfect process design features will resolve them.

Bringing together academic, civil society, private sector, and governmental organizations, transdisciplinary collaborations bring institutional values and cultures into stark relief. In CARIAA, we have encountered many of the challenges associated with this key systemic barrier. These include differences in commitments and abilities to meet deadlines, differences in staff time allocations to projects between institutions, and the growing pressure on academic staff to produce high impact publications at all costs, including at the cost of pursuing other types of impact through collaborative efforts.

Navigating these deep systemic barriers has become a full‐time occupation for a key type of actor in climate change research: consortium coordinators, who coordinate rather than do the research, and if effective, add significant value. Consortium coordinators play perhaps the most central role in this new genre of large‐scale research projects. Effective coordinators go beyond traditional management roles, mediating between institutions with different values, being attentive to power asymmetries, and working to navigate systemic barriers such as those embodied in grant agreements. However, consortium coordinators cannot substitute the need for strong project leadership, and indeed may become disabled by the lack of support from a hands‐on leader. Project coordinators and research leaders thus emerge as a key unit for success in large‐scale collaborative research programs.

Despite the relational and systemic constraints described here, the emergence of a handful of self‐organizing, unplanned, and highly successful collaborations in CARIAA offer insight into factors that help transcend these constraints.

In the first case, a tight knit group drawing from the four consortia has developed around pursuing research impact, and has worked collectively to share lessons about effective approaches, coordinate activities, and to develop joint products of common interest. As a self‐selected and voluntary group, barriers around trust have largely been avoided. Other key ingredients for this group have been a shared and codeveloped language around which to share experiences, and a shared interest in what they are doing. In other words, this group has emerged as a community of practice.^20^ Some design elements have facilitated this group's collaboration, including the flexibility in the program management design which allowed for funds to be mobilized to support their interests. The ability to provide funds for face‐to‐face meetings when the demand arose was crucial. Efforts to sustain this group using virtual tools such as a shared intranet space have not been particularly successful. This experience suggests that although communities of practice can emerge in dispersed and large‐scale collaborative efforts, leadership, and resources are required to ensure opportunities for face‐to‐face interaction and to create the enabling environment for these shared interests to be discovered.

Another strong community of practice emerged in one consortium around the topic of migration. The circumstances in which this collaboration emerged could not have been planned for, although some design elements made a key difference. The collaboration was facilitated by a flexible small grant, again highlighting the importance of such flexible mechanisms within large‐scale transdisciplinary teams. However, timing was just as important: the grant became available at a moment in the life span of the project when the researchers involved already had good initial relationships and trust in each other's abilities. Furthermore, each of the researchers was able to commit to the research, and there was a willing champion of the group who played a convening function. Importantly, the focus of the work fitted into existing agendas and commitments of the individuals, their institutions, and the broader program. This gave the group a sense of endorsement and encouragement that maintained enthusiasm.

In both the examples of communities of practice, a facilitator, or champion, was a key feature. Timing was also critical, as both the groups emerged close to half way through the program and were unplanned at the outset. Both also depended on the availability of flexible funds to enable them to capitalize on their common interests.

Environmental challenges such as climate change are urgent, highly complex, multiscale, and wicked. To tackle challenges with these characteristics, transdisciplinary collaborations that are commensurate with the scale of the challenge, learning‐oriented, and problem‐focused are necessary. However, for donors preparing to fund large‐scale collaborative efforts, and for researchers submitting proposals for such endeavors, we recommend proactive steps, up‐front, to identify and, where possible, address all three layers of collaborative endeavor highlighted here, from design to systemic features of transdisciplinary collaboration (Figure 2). In the arena of climate change adaptation research, and indeed global environmental change research more generally, it is no longer sufficient to approach transdisciplinary collaboration purely from a design perspective. Paying attention to the barriers to collaborative endeavor in transdisciplinary settings is essential.^15^ Our learning shows that dedicated consortium coordinators, leaders at multiple levels, timing and the availability of small amounts of flexible funds are all crucial, indicating a new kind of agility and nimbleness necessary to achieve successful outcomes.

## Conflict of Interest

The authors declare no conflict of interest.
